# To See and Be Seen: A Swedish Register Study on Children Who Witness Family Violence

**DOI:** 10.3390/ijerph21101291

**Published:** 2024-09-27

**Authors:** Sven Trygged, Tove Bylund Grenklo, Anneli Marttila, Niklas Halin

**Affiliations:** 1 Department of Social Work, Criminology and Public Health Sciences, Faculty of Health and Occupational Studies, University of Gävle, 80176 Gävle, Sweden; anneli.marttila@hig.se; 2 Department of Caring Sciences, Faculty of Health and Occupational Studies, University of Gävle, 80176 Gävle, Sweden; tove.bylund.grenklo@hig.se; 3 Department of Occupational Health, Psychology and Sports Sciences, Faculty of Health and Occupational Studies, University of Gävle, 80176 Gävle, Sweden; niklas.halin@hig.se

**Keywords:** family violence, report-of-concern, social services, Sweden

## Abstract

Authorities and civilians can make a report-of-concern to Social Services if they suspect a child is experiencing or witnessing violence. In 2021, Sweden implemented new legislation that considers children as victims of crime not only when abused but also when witnessing family violence, i.e., Barnfridsbrott. This study aimed to describe and analyze reports-of-concern regarding children witnessing family violence. Are there any changes in number of reports over the years? Who is reporting? And what interventions are most frequent? This is a register-based study of reports-of-concern in Gävle municipality in Sweden for the years 2018–2022. This unique register makes it possible to identify and follow up reported cases as long as they are active by Social Services. Results show there was already a major increase in the number of reports-of-concern in 2020. Most reports are made by Social Services and the police. Of all reports related to family violence, an increasing number lead to further investigations. In most of those cases, the children stay with their families, and Social Services offer counselling. Conclusion: there was a distinct increase in reports related to children witnessing family violence already starting in 2020 in the studied municipality, before the new legislation was implemented.

## 1. Background

Experiencing or witnessing violence within the family is among the most difficult things a child can endure and is associated with extensive suffering both in the short and long term. Children who are exposed to, or witness, violence within the family may need significant acute support and protection. Witnessing violence between family members also puts children at an increased long-term risk of developing anxiety, depression, PTSD [1,2], substance abuse, and self-harm, as well as facing challenges in academic performance, memory, and verbal abilities, and facing an increased risk of being bullied [2]. This also risks entering relationships where the victims are subjected to or perpetrate violence. It is also known that a household’s dysfunction may lead to premature death in adulthood [3]. Known risk factors for perpetrators to reoffend in violence include substance abuse, controlling and dominant behavior, jealousy, threats, and desires for divorce [4,5,6,7]. Violence can have both direct and indirect consequences. Trygged et al. have, in studies of women [8,9,10] and young men [11] who have been subjected to violence, highlighted economic difficulties and mental health problems among the victims.

The suffering of children in environments where violence occurs can be assumed to be universal. In Western countries, social authorities are expected to act when children are in such environments. However, legislation, types of intervention, and the treatment of children vary from country to country. In this study, data from one municipality in Sweden are used, and the study is limited to this municipality in Sweden and Swedish conditions.

### 1.1. The Swedish Context and Previous Research from Sweden

According to the Convention on the Rights of the Child (CRC), which Sweden ratified in 2020, the best interests of children should be considered. What is in the best interests of the child is a question of interpretation, which is a complicating factor in practice. The Ombudsman for Children in Sweden is a government agency tasked with promoting and advancing children’s rights and interests in Sweden on the basis of the CRC. The authority argues that the principle of the child’s best interests consists of three parts: a substantive right, a legal principle of interpretation, and an approach in decision-making processes [12]. Children have the right to life, survival, development, to be heard, and to be protected from violence, neglect, and abuse, including sexual abuse. A Swedish overview of the current knowledge shows that it is common for children themselves to be subjected to physical violence when living in a family where one parent abuses the other [13].

The National Board of Health and Welfare issued advice to social services in 2014 to pay attention to children who witness violence [14]. Several years later, it was estimated that around 210,000 children in Sweden were living in homes where various forms of violence occurred [15]. In a national survey study of approximately 4700 students aged 15 and 17, 14 percent reported having witnessed violence in the family at least once during their lifetime [16].

Since 2021, it has been a criminal offense to expose children to violence, threats, or abuse between family members, known as Barnfridsbrott (violation of a child’s integrity; BrB 4 chapter, 3 §). This offense is part of criminal justice and was not followed by any directed means to Social Services. In October 2023, for the first time, compensation for severe Barnfridsbrott was granted when two affected children received the same amount of compensation as their mother, who was severely assaulted in front of them [17].

**Figure 1:**
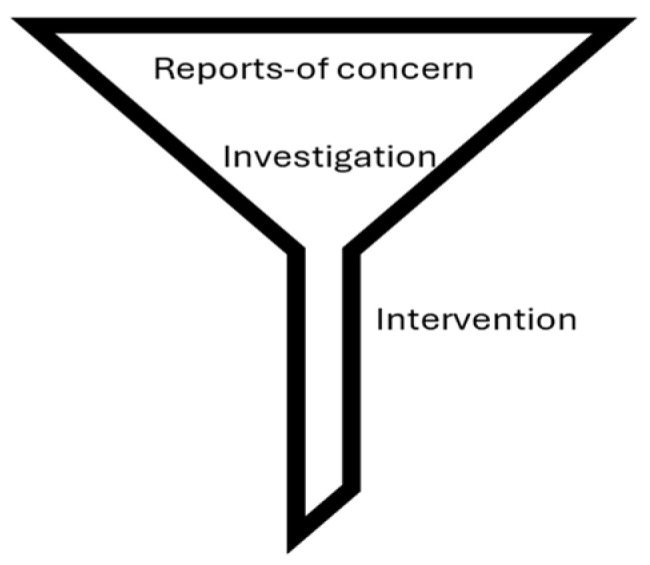
The child welfare funnel.

Social Services are society’s safety net for children in need of protection and support. Social Services typically become aware of children at risk when someone submits a so-called report-of-concern regarding the child in question. Employees of certain authorities, as well as their private counterparts, such as preschools, schools, and healthcare facilities, are obligated by the Social Services Act [18] to report to Social Services if they suspect that a child or adolescent is at risk. Private individuals can also file reports-of-concern. When a report-of-concern is received by Social Services, an assessment of the situation is made. This may involve contacting the person who made the report, contacting the child, and other relevant individuals to gather information. The social worker decides whether an investigation should be initiated. If an investigation is conducted and shows a need, Social Services may decide on interventions. Sometimes, emergency interventions are required, such as sheltered accommodation or temporary care. Other interventions may include parenting support, housing support, or support for the child at school. Interventions for children at risk can be carried out voluntarily under the Social Services Act (SOL) or, in more severe cases, by coercion under the Care of Young Persons Act (LVU). The process itself is sometimes described as a “child welfare funnel” (see Figure 1) where many children are initially involved (reports-of-concern), but fewer are investigated and even fewer receive interventions (see, for example, [19,20]).

Previous research in Social Services points to significant heterogeneity regarding both case handling, attitudes towards issues affecting case handling, and the choice of sanctions (see, for example, [19]). Social Services are required to report suspicions of children being subjected to violence to the police [14].

In a literature review [21] regarding risk assessment, safety planning, and support for children exposed to violence in the family, four risk areas were identified: when there are threats to (a) the children’s safety (e.g., if they intervene and try to protect their mother or siblings); (b) the children’s reactions; (c) the children’s perspective in terms of fear and helplessness; and (d) the children’s development (e.g., due to the loss of a secure parent, post-traumatic stress, and inability to comprehend what is happening).

Recent research, however, suggests that despite the new law on offenses against children’s integrity, the threshold for support and protection to be provided for the children, even when strong warning signs are present, remains high [22]. According to Ponnert [23], among others, this is mainly because the parents do not consent.

In a review of 264 child welfare investigations from 12 municipalities in Sweden, it was shown that almost 50% of the parents had declined or otherwise obstructed Social Services’ interventions, for example, by stating that the problems do not exist or are no longer relevant. Social Services rarely provided the children with the opportunity to be heard in individual sessions [24]. The parents’ lack of consent thus infringes upon the children’s rights. Factors that complicate the victim, often the mother, from daring to report her partner include, for example, the presence of threats of deadly violence, lack of social support, substance abuse, and/or economic problems [25], which in turn simultaneously diminishes her ability to protect her children. Furthermore, risk assessments for the children are rarely conducted when they are not the primary victims [26]. In a recently conducted literature review, it was shown that secure circumstances; reliable and accessible caseworkers; comprehensible information about what is happening; the child’s ability to choose what, if, and when it wants to speak; being treated with respect; engaging in activities and having fun; and not just being treated as a victim are perceived as valuable support by children who have been exposed to violence in the family [27].

One reason why the more systematic knowledge in the field is limited may be due to the fact that municipalities’ data registration is designed for administrative needs and not for research. National documentation systems are missing. A difficulty in this context is to provide evidence that children have witnessed violence since few outsiders were present when the violence occurred. It is therefore important to find out who makes reports-of-concern (specifically for witnessed violence) to Social Services and what happens afterward.

If Social Services should work from the intentions in Agenda 2030 (no one left behind) referring to “equity and social justice” [28], we need to get deeper knowledge and to follow up also on sex, age, and socioeconomic and other important factors related to the child. This knowledge can be used to further develop successful interventions for children and their families, but also to prevent violence in the family context. Finally, a survey from Finland showed that girls reported having witnessed all forms of intimate partner violence against both parents much more often than boys did. The authors speculate if these differences depend on real differences or if boys and girls interpret violence differently [29]. Since we have data on reports-of-concern for both boys and girls, we could study if there is such difference in the actual cases known by Social Services.

### 1.2. Purpose and Research Questions

The overarching aim is to increase and deepen systematic knowledge about social child welfare support and interventions for children where concerns have been reported about witnessing violence within the family.

#### Research Questions

- Who makes the reports-of-concern about violence in close relationships involving children to Social Services?
- Has there been an increase in reports-of-concern concerning children witnessing violence since the new legislation Barnfridsbrott (violation of a child´s integrity) was introduced?
- What interventions and support do Social Services provide when reports are made about children witnessing or being subjected to violence in their home environment?
- Are there any differences in reported cases and means of support between boys and girls of different ages?

## 2. Methods

We use register data from Gävle municipality called “Violence in the family” which is related to violence between family members, i.e., where the child is forced to witness (see or hear) violence in his/her immediate environment. A strong limitation is that we cannot tell from register data who the perpetrator is or if violence takes place in a certain stage of the relationship between the adults (c.f. [30]).

Some children may be reported more than once by different authorities and others. However, due to ethical and practical limitations, we do not have register data for individual children. Thus, in our analysis, it was not always possible to separate the number of children from the number of reports. Therefore, in the analysis we report the number of reports-of-concern, and not the number of unique children.

Through an analysis of these population-based register data, knowledge about the conditions of vulnerable children and about society’s efforts, as well as what predicts the choice of intervention, can increase. Register data can be used both to explain, describe, evaluate, and find forms of preventive work. The project is based on population-level register data from Gävle municipality, a municipality with about 100,000 inhabitants (a medium-sized town in the Swedish context). Gävle municipality has systematically collected information about children who encounter Social Services due to reports-of-concern (suspicion that something is not right). The municipality’s register data contain information about who makes the report and the reason for the report. It is possible to break down the material and study the occurrence of reports-of-concern within several sub-areas. There are a wide range of reasons for reports-of-concern, among which the most serious may involve abuse and the presence of violence in the family. Data are available for the period 2018–2022.

### Ethical Considerations

Register data from Gävle municipality are provided in an anonymized form, ensuring individual privacy. A data management plan has been developed in consultation with the IT department at the University of Gävle, outlining data storage and access procedures. The study has received ethical approval from the Swedish Ethical Review Board (Dnr 2023-00622-01).

## 3. Results

**Figure 2:**
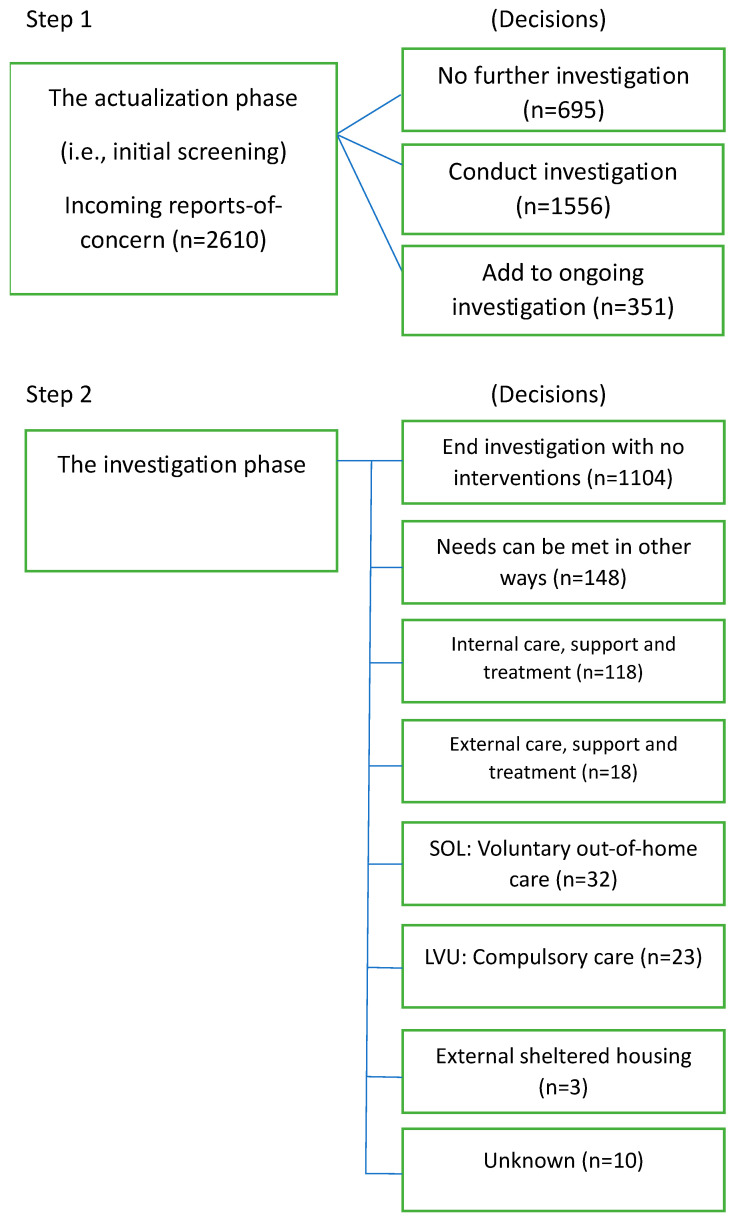
From report-of-concern to intervention. Flowchart. Gävle municipality years 2018–2022. Note: There is a discrepancy between the number of investigations initiated (i.e., 1556) and the total number of decisions made (i.e., 1456). Possible explanations for this include ongoing investigations where decisions have not yet been made, potential errors in registration, or lost data.

The Results section starts with the “child welfare funnel”. Figure 2 shows an overview of how Social Services have dealt with the received reports-of-concern for 2018–2022 in terms of frequency of data. Each segment will be explained more thoroughly further below.

### The Number of Reported Cases

During the five-year period between 2018 and 2022, Social Services handled a total of 2610 reports-of-concern regarding domestic violence (see Figure 2).

**Figure 3:**
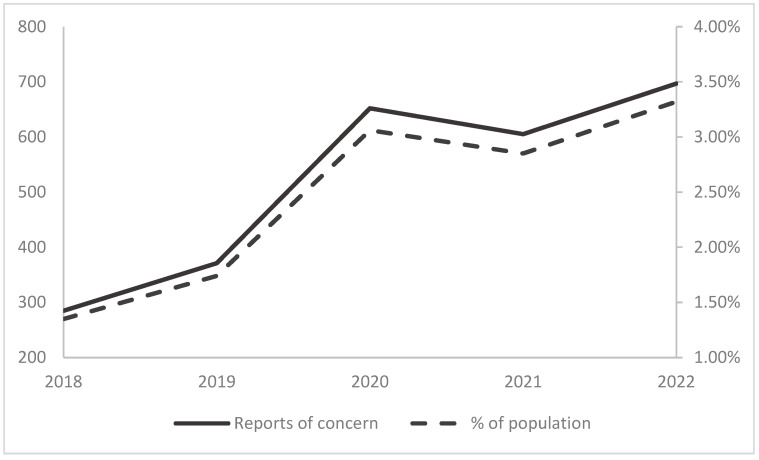
Overall picture of reported cases of concern in Gävle municipality 2018–2022.

As Figure 3 (primary axis) shows, there was an increase of reports-of-concern between 2018 and 2022. Looking at all reported cases, there were 144.6% more reports-of-concern in 2022 compared to 2018. A one-variable *Χ*^2^ showed that the observed frequency distribution was significantly different compared to an equal distribution during the five-year period (2018–2022); *Χ*^2^(4, N = 2610) = 255.53, *p* < 0.001. The largest increase between two years was between 2019 and 2020, with 75.7% more reports in 2020.

In order to investigate if and how the patterns coincide with the number of reported children and the proportion of children in Gävle living in households involved in reports-of-concern, the number of reports were divided with population data (see Figure 3, secondary axis). During the five-year period, the ratio between reports-of-concern and the number of children increased from 1.35% in 2018 to 3.13% in 2022, whereas the largest increase occurred between 2019 and 2020, with an increase of 1.29 percentage points.

Figure 3 shows the number of reports-of-concern involving a child (primary axis) in Gävle municipality and the proportion of all cases of reports-of-concern in the population of children between 0 and 17 years in the same municipality (secondary axis).

1. Who is reporting?

Social Services and the police were the two most frequent reporters, responsible for 66.6% of all reports during the period from 2018 to 2022, where Social Services were the largest contributor with a total of 1070 reports (see Table 1). That the observed frequency distribution between the reporters was significantly different from an equal distribution was supported by a one-variable *Χ*^2^; *Χ*^2^(6, N = 2610) = 2359.96, *p* < 0.001. During this five-year period, there was also an increase in how many of the reports-of-concern led to further investigation by Social Services (i.e., either a new investigation or added to an already ongoing investigation). A one-variable *Χ*^2^ showed that the observed frequency distribution (i.e., the proportion of cases that were further investigated) was significantly different from an equal distribution; *Χ*^2^(4, N = 347) = 18.55, *p* < 0.001. This significant difference in frequency distribution is due to the low proportion of cases that were further investigated in 2018 compared to 2019–2022. There is also a notable difference between the reporters concerning the proportion of the reports-of-concern that led to further investigation. With reports from parents or caregivers (87.5%), Social Services (82.6%), and preschool (80%) leading to a higher proportion of investigations than school (66.7%), healthcare (68%), and police (67.3%). However, a one-variable *Χ*^2^ could not support that the observed frequency distribution was different than an equal distribution; *Χ*^2^(5, N = 453) = 5.74, *p* = 0.332.

2. What interventions do Social Services offer?

During the period 2018–2022, approximately 73% of the 2610 reports-of-concern led to further investigation, and of these 195 (approx. 10%) were deemed to require some sort of intervention. As can be seen in Table 2, the most frequent types of interventions were the ones belonging to the category *Internal care, support and treatment* (e.g., counselling, crisis counselling), which was used 60.5% of the time, followed by *SOL: Voluntary out-of-home care* (e.g., residential care, foster care; 16.9%), *LVU: Compulsory care* (e.g., temporary residential/foster care, sheltered housing; 11.8%), *External care, support and treatment* (e.g., intensive family support, parental counselling; 9.2%), and *External sheltered housing* (1.5%). The interventions belonging to the category *Internal care, support and treatment* were the most common. This is supported in the analysis by a one-variable *Χ*^2^ that showed that the observed frequency distribution was significantly different from an equal distribution; *Χ*^2^(4, N = 179) = 243.60, *p* < 0.001. Looking at separate intervention types, different kinds of counselling were by far the most used intervention (approx. 62%), followed by crisis counselling (approx. 5%) and intensive family support (approx. 5%) (See Figure 2).

3. Minor differences between boys and girls

Boys (∑ = 1337) were slightly more frequent in the reports-of-concern compared to girls (∑ = 1237 reports (see Table 3). Moreover, most reports were in the age group 6–12 years (∑ = 1208), compared to 0–5 years (∑ = 866) and 13–17 years (∑ = 536). However, a 3 (Age group) × 2 (Gender) independence *Χ*^2^ showed that there was no significant association between the two factors; *Χ*^2^ (2, N = 2610) = 4.65, *p* = 0.098.

Looking at gender and the type of intervention implemented, descriptive data for boys and girls look rather similar (see Table 2), which is supported by a 4 (Type of intervention) (*External sheltered housing* has been excluded prior to the analyses due to the low number of observations) × 2 (Gender) independence *Χ*^2^, which showed no significant association between the two factors; *Χ*^2^ (3, N = 191) = 3.18, *p* = 0.364. Regardless the lack of significant association, one difference is notable, with *External care, support and treatment* used 2.6 times more for boys than girls.

The overall picture shows there are almost no differences in the number of reports-of-concern between boys and girls when it comes to children witnessing violence. The interventions implemented by Social Services also seem to be almost the same. There were more reports referring to children ages 6–12-years-old compared to both younger and older age groups, but the pattern for which intervention was offered to boys and girls was similar.

## 4. Discussion

Register data can be used both to explain, describe, evaluate, and find forms of preventive work. This work also aligns well with the investigation Sustainable Social Services [31], which emphasizes the importance of developing Social Services statistics.

This study concerns both children witnessing family violence and the importance of identifying and supporting these children. This study aims also to find ways to track the process from report-of-concern to its conclusion (the “child welfare funnel”). Register data enable the study of whether there are differences in the process depending on who makes the report. By reviewing and processing this data, it becomes possible to create both a better overall picture and deeper knowledge regarding reports-of-concern where children witness violence related to the home and/or a specific area, such as preschool/school. Gävle municipality’s register enables monitoring of all children for whom reports-of-concern were made between 2018 and 2022. This can be considered unique data.

The main aim of the study was to describe and analyze reports-of-concern with a focus on family violence and children before and after the new legislation on violation of a child´s integrity. Are there any changes in the number of reports? By whom they are reported? And what interventions are predominant?

Some results are quite clear. Many actors make reports-of-concern about children in need, but most cases referring to family violence are reported from the police and from Social Services (including the department of social emergency). The number of reported cases increased during the study period 2018–2022. At the same time, there was also a strong increase in the proportion of investigated cases. This coincides with the new legislation, but not completely. The results showed that there was a sharp increase in the number of cases as early as 2020, i.e., the year before the new law Barnfridsbrott was implemented in Sweden. However, the Convention of the Right of the Child (CRC) was enforced in Swedish legislation in 2020. It is possible that both the police and Social Services had started adapting routines to one or both laws (since the reporting of these organizations increased the most). However, the reporting from preschools and schools, organizations that regularly meet children, also increased but not as much as the police and Social Services, which may go against a general awareness related to the implementation of the CRC. Perhaps there is an underreporting from preschools and schools. Another explanation may be the pandemic. The pandemic greatly reduced contacts and activities, so perhaps there were more conflicts in families resulting in violence. It is also difficult to know if the authorities were more alert and reported more. Additionally, the pandemic resulted in lower employment rates and higher unemployment [32], which put more financial strain on some families. Still there are more changes that are difficult to fully explain. The increase in not only the number of reported cases but also an increase in the proportion of investigated cases. This may reflect policy changes, possibly the new forms of legislation, but this is something that needs to be studied further.

One research question was about what kind of support Social Services offers. Most support was based on voluntary agreements (through the Social Services Act), and some support was forced upon the child when conditions were severe. The most common forms of voluntary support were different forms of counselling.

The final research question was about comparing reports to Social Services for boys and girls in different age groups. Are the numbers equal, and do both sexes receive the same kind of support?

The fact that there was no difference between boys and girls witnessing violence may not be surprising. Most likely, in almost all cases, for example, violence between couples does not depend on the sex of the child(ren). As there are about the same number of boys and girls in the population, about the same number of boys and girls may witness violence. A recent Finnish survey by Hietamäki et al. [29] found that girls were more exposed to witnessing violence than boys. Our findings from actual reports-of-concern do not support their results. One key to understand this discrepancy may be a study by Kalin et al., which showed that most children who self-rated themselves as severely exposed were never referred to Social Services [33]. Other research has pointed at the importance of the child’s social background and type of violence [34]. The authors found, for example, that exposure to physical violence was more common among boys living in higher-status areas, whereas neglect was more common among children living with only one parent of lower income. However, as we do not know if there are differences in how boys and girls respond to domestic violence, it is difficult to evaluate if the interventions are satisfactory. We can only conclude that boys and girls receive the same interventions as reported by Social Services, according to the studied register data. Regarding the ages, the most frequent reported violence is found among children 6–12-years-old. We lack knowledge if this depends on the possibilities for children to express themselves (which of course is difficult for the youngest children) and changes in family constellations. Since we know already from earlier research [35] that if the violence occurs when the child is very young, it has different effects than if it occurs when the child is older, we intend to examine these ambiguities in forthcoming interview studies with social workers.

### Limitations

The study is limited to one municipality in a Swedish context. Legislation, interventions, and treatment approaches may vary in other countries and contexts. Additionally, the short time span of available data limits the ability to draw conclusions about the impact of recent legislative changes. Since the law was enforced in 2021 and our latest data come from 2022, it is too early to know if this is a temporary or permanent increase. If the increase in reports-of-concern is temporary, it may reflect the pandemic, and if it is permanent, it may rather reflect the policy changes. One more limitation in the study is that we could not study family structure or socioeconomic factors related to reports-of concern, since socioeconomic factors have shown to be important in understanding the distribution of support from Social Services [34].

## 5. Conclusions and Further Research

The main conclusion is that there was a distinct increase of reports related to children witnessing family violence, starting already in 2020, before the new legislation was implemented.

For future research, it would be highly valuable to obtain firsthand information from the stakeholders, the children themselves. However, this is a quite complicated undertaking with many ethical and practical difficulties. Another research strategy could be to involve staff, and for future research, it could be interesting to discuss register findings with staff from Social Services. Staff can reflect on the children’s situation related to reports-of-concern for children who witness violence and the interventions that are made, and they can also help interpret the results. By gaining a better understanding of the interventions that are made, it may be possible to understand which interventions seem supportive for the child (at different ages) and what distinguishes the more “successful cases”. This information could be relevant for prioritizations and working methods related to children and parents. It is not entirely certain that the cause of the report or investigation itself determines the intervention. What likely determines the intervention more is the degree of vulnerability of the child. The more serious the situation is for the child, the greater the risk of placement. These are examples of questions that can be highlighted in future research and discussed to contribute to knowledge that can further form the basis for interventions for the target group.

## Figures and Tables

**Table 1:** The number of reports-of-concern of domestic violence involving a child between the years 2018 and 2022.

Reporter	2018		2019		2020		2021		2022		Total	
	Number of Reports	% Leading to Investigation	Number of Reports	% Leading to Investigation	Number of Reports	% Leading to Investigation	Number of Reports	% Leading to Investigation	Number of Reports	% Leading to Investigation	Number of Reports	% Leading to Investigation
Social service *	76	42.1	163	76.1	278	85.3	246	90.2	307	87.6	1070	82.6
Police	97	32.0	98	56.1	164	73.8	152	77.6	156	79.5	667	67.3
Healthcare	20	50.0	9	77.8	35	62.9	36	80.6	25	68.0	125	68.0
Preschool	6	0.0	16	75.0	26	80.8	22	90.9	25	92.0	95	80.0
School	12	33.3	15	60.0	18	77.8	27	81.5	30	63.3	102	66.7
Parent/caregiver	17	76.5	19	89.5	17	88.2	16	100	11	81.8	80	87.5
Others	57	36.8	51	52.9	114	65.8	106	73.6	143	58.7	471	60.5
Total	285	39.0	371	67.8	652	77.5	605	83.5	697	78.2	2610	73.5

* Incl. social emergency services. N = number of reports-of-concern and the percentage leading to investigation broken down by reporter years 2018–2022.

**Table 2:** The number of interventions used by Social Services broken down by main category of interventions between the years 2018 and 2022 for girls and boys, respectively.

Type of Intervention	Girls	Boys
Internal care, support, and treatment	59	59
SOL: Voluntary out-of-home care	16	16
External care, support, and treatment	5	13
LVU: Compulsory care	11	12
External sheltered housing	2	1
Total	93	101

**Table 3:** The number of reported cases of domestic violence involving a child broken down by age group and gender between the years 2018 and 2022.

Age Group	2018		2019		2020		2021		2022		Total	
	Girls	Boys	Girls	Boys	Girls	Boys	Girls	Boys	Girls	Boys	Girls	Boys
0–5 years	42	61	66	62	95	116	77	110	118	119	398	468
6–12 years	69	57	93	85	132	161	165	118	140	188	599	609
13–17 years	30	26	39	26	62	86	71	64	74	58	276	260
Total	141	144	198	173	289	363	313	292	332	365	1273	1337

## Data Availability

Data is unavailable due to ethical restrictions.
